# Estimating Prevalence of Illicit Drug Use in Yunnan, China, 2011–15

**DOI:** 10.3389/fpsyt.2018.00256

**Published:** 2018-06-14

**Authors:** Guanbai Zhang, Heng Jiang, Jiucheng Shen, Pinyuan Wen, Xuebing Liu, Wei Hao

**Affiliations:** ^1^Key Laboratory of Psychiatry and Mental Health of Hunan Province, China National Clinical Research Center for Mental Health Disorders, Mental Health Institute of the Second Xiangya Hospital, National Technology Institute of Psychiatry, Central South University, Changsha, China; ^2^Yunnan Institute for Drug Abuse, Kunming, China; ^3^Centre for Alcohol Policy Research, School of Psychology and Public Health, La Trobe University, Melbourne, VIC, Australia; ^4^Centre for Health Equity, Melbourne School of Population and Global Health, University of Melbourne, Melbourne VIC, Australia; ^5^Affiliated Wuhan Mental Health Center, The Ninth Clinical School, Tongji Medical College of Huazhong University of Science and Technology, Wuhan, China

**Keywords:** prevalence, illicit drug use, estimation of population size, capture-recapture method, Zelterman's estimate, heroin, methamphetamine, China

## Abstract

**Background and Aims:** Yunnan has been one of the provinces most afflicted by illicit drug use in China. However, there have been few systematic studies on the prevalence of illicit drug use in the area.

**Method:** Using data on illicit drug users registered in the police system, this study aims to estimate the population size of illicit drug users and the annual prevalence of drug use in Yunnan from 2011 to 2015 using Zelterman's capture-recapture method.

**Results:** In the 15–64 year-old population, the estimated prevalence rate of any illicit drug use was 0.81% (0.76–0.86%) in 2011 and 0.94% (0.90–0.98%) in 2014, representing a significant increase. The prevalence rate of heroin use decreased from 0.67% (0.63–0.73%) in 2011 to 0.57% (0.53–0.61%) in 2015, while the prevalence rate of methamphetamine use doubled from 0.20% (0.17–0.24%) in 2011 to 0.48% (0.46–0.50%) in 2015. The mean age of the 141,702 identified illicit drug users in Yunnan from 2011 to 2015 was 32.2 years, and the majority were male, low educated, and peasants; nearly half were single and ethnic minorities. The ratio of the number of identified illicit drug users to the number of hidden users increased from 1:12 in 2011 to 1:5 in 2015.

**Conclusion:** The prevalence of illicit drug use in Yunnan has been increasing. Although the prevalence of heroin use decreased, methamphetamine use increased dramatically from 2011 to 2015. Preventive strategies that are more effective in reducing illicit drug use are urgently needed in Yunnan.

## Introduction

Illicit drug use can cause many negative consequences, including epidemics of HIV/AIDS and hepatitis C, cardiovascular disease, fatal or nonfatal overdose, intentional or unintentional injuries, crime, and mental disorders (1). Comorbidity of mental disorders in substance users is related to its high prevalence, clinical and social severity, difficult management, and association with poor outcomes for affected subjects (2), as well as long illness course (3, 4), high rates of disability, morbidity, and treatment non-adherence (5). Among drug users, depressive disorders are associated with non-fatal overdose (6). Globally, drug use accounted for 20 million disability-adjusted life years (DALYs) in 2010 and 28 million DALYs in 2015, due to premature death and disability (7, 8).

Bordering the notorious “Golden Triangle” and with a population of about 47 million^1^, Yunnan has been one of the areas most afflicted by illicit drug use in China. The seizure of heroin and methamphetamine (MA) in Yunnan accounted, respectively, for about 80 and 70% of the total seizures in China during the third national War on Drugs (2011–15)^2^. With 188,000 registered illicit drug users (as of May 2015), Yunnan ranked fifth in 31 Chinese mainland provincial regions. In addition, of 39,472 clients enrolled in methadone maintenance treatment (MMT), 20,777 were being treated in Yunnan in 2014^3^ (9).

However, besides reports of official data from the judicial system and treatment system, there have been few systematic studies on the extent of illicit drug use. This study aims to fill this gap by estimating the annual prevalence of illicit drug use in Yunnan between 2011 and 2015. Annual prevalence has been adopted as one of the key indicators to measure the extent of illicit drug use (10).

Unlike tobacco smoking or alcohol consumption, the use of illicit drugs such as heroin or MA is prohibited in many countries. This makes it difficult to determine the extent of its use through a traditional epidemiological survey, as there is no sampling frame and drug users often deny their use. Consequently, the population size of illicit drug users is usually estimated by indirect methods, such as census/enumeration, the capture-recapture-method (CRM), network scale-up, or a multiplier (11). The CRM was derived from ecology, and has been increasingly used for estimating the population size of drug users and the prevalence rate in recent years (12–17). In this study, the CRM was used to estimate the population size and prevalence of any drug use, heroin use, and MA use in Yunnan between 2011 and 2015.

## Materials and methods

### Materials

The registers of the Dynamic Management and Control System for Illicit Drug Users from 2011 to 2015 were used in this study. The system was developed by the Chinese Ministry of Public Security in 2004, and became operational in 2008. Under the Anti-Drug Law of the People's Republic of China (18), anyone whose illicit drug use is verified will be registered in this system. The details recorded comprise: date of identification; personal information, including name, national identification (ID) number, sex, date of birth, ethnic group, education level, employment, marital status, and address; and details of the drug use (e.g., name and source of the drug). A mug shot of the user is also captured and stored in the system.

Only police officers have access to the system and its data. The data we used in this research were encrypted and provided by the Chinese Ministry of Public Security. Nationwide, there were 6.5 million records (i.e., 6.5 million identifications) from January 1, 2008 to June 30, 2016.

A drug user can be identified only once per day. If a drug user had two or more identification records for a given day, we considered only one to be valid and disregarded the other(s) as invalid. Except for repeat identification record(s) on the same day, there were no other implausible data. Of 224,901 records of illicit drug user register in Yunnan from 2011 to 2015, 2,699 (1.2%) were considered invalid; the remaining 222,204 (98.8%) were included in the analysis.

Each identified drug user is assigned a unique number by the computer system when registered for the first time. Using the unique number, the total number of identified drug users in each year can be calculated. The number of drug users caught once or multiple times can also be respetively calculated.

### Zelterman's estimator

In a given time period, a drug user in the community can be identified or not by the police, For the identified ones, some are identified only once, some twice, some three times or more. According to the identification count of each drug user, special statistical techniques can be used to estimate the population size of drug users.

Zelterman's estimator (19) is an ideal method to estimate population size with this kind of data (17). First, it is simple and estimates the hidden population with only a low rate of exposed cases. The model emphasizes the lower frequency classes, based on the assumption that drug users who are rarely identified have a greater similarity than often-identified users to those who are never identified. According to Zelterman and other researchers (17), the models should be complex enough to be meaningful, but simple enough to contain only the parameters that are necessary, and close to the quantity to be estimated. Second, this model has narrow 95% confidence intervals (CIs), because the greater the range, the larger the level of uncertainty of the estimate (17). Zelterman's estimator (19) can be written as follows:

$$\begin{array}{r} N_{all}=\frac{N_{iden}}{1-e^{\left(-2\times f_{2}\right)/f_{1}}} \end{array}$$

where *N*_*all*_ is the estimate of total population size; *N*_*iden*_ is the total number of identified users in the given time period; *f*_1_is the number of drug users identified only once; *f*_2_ is the number of users identified twice; and *e* is the base of natural logarithms (*e* = 2.71828). MySQL and SPSS were used to read and analyze our data. Besides the CRM, one-way ANOVA and Chi-square statistics were used to test the demographic difference of drug users across the five years of the study period, with a significance level of 0.05. Missing data were excluded from the analysis (e.g., missing values in occupational and marital status). The missing values were smaller less than 1% of the total sample size had missing values, so these are unlikely to impact our analysis.

## Results

### Status of identified drug users in Yunnan, 2011–15

Various types of illicit drugs have been used in Yunnan, including heroin, opium, MA, methylenedioxymethamphetamine (MDMA), ketamine, marijuana, benzodiazepine, cocaine, tramadol, etc. In total, 141,702 illicit drug users were identified by the police between 2011 and 2015.

The two most commonly used drugs were heroin (90,766 identified users) and MA (56,159 identified users), accounting for 97.3% of all the identified drug users. The number of identified drug users increased (128.4%) from 21,638 in 2011 to 49,411 in 2015. Heroin users increased (62.4%) from 17,179 in 2011 to 27,907 in 2015, while MA users increased almost fourfold (398.5%), from 4,459 to 22,229 (see Table 1 for more details).

**Table 1:** The numbers of main illicit drug users identified in Yunnan (2011–15).

	**Heroin**		**MA**		**Both heroin & MA**		**Ketamine**		**Marijuana**		Other drugs^*^		**Total (Any drugs)**
	***n***	**%**	***n***	**%**	***n***	**%**	***n***	**%**	***n***	**%**	***n***	**%**	***n***
2011	17,179	79.39	4,459	20.61	1,029	4.76	63	0.29	11	0.05	39	0.18	21,638
2012	20,538	72.24	8,324	29.28	1,831	6.44	51	0.18	18	0.06	48	0.17	28,430
2013	23,367	67.38	11,802	34.03	2,314	6.67	27	0.08	23	0.07	80	0.23	34,679
2014	28,642	64.15	16,368	36.66	3,146	7.05	37	0.08	55	0.12	98	0.22	44,646
2015	27,907	56.48	22,229	44.99	3,560	7.20	100	0.20	111	0.22	132	0.27	49,411
Total^a^	90,766	64.05	56,159	39.63	11,291	7.97	274	0.19	217	0.15	396	0.28	141,702

* *Other drugs include MDMA, benzodiazepine, cocaine, tramadol, etc*.

a *The total number of users identified during the five year period (not the sum of the annual numbers of identified drug users)*.

### Descriptive statistics of identified drug users in Yunnan, 2011–15

Characteristics of the illicit drug users identified between 2011 and 2015 are shown in Table 2. The mean age was 32 years, and over 91% of identified illicit drug users were male. Other notable findings include the following: more than half were Han nationality (57.5%); nearly half (46.8%) were primary school graduates or below; half (50%) were unmarried; and more than half (58.5%) were peasants.

**Table 2:** Demographic characteristics of identified drug users in Yunnan (2011–15).

	**2011**		**2012**		**2013**		**2014**		**2015**		**2011–15**			
	***n***	**%**	***n***	**%**	***n***	**%**	***n***	**%**	***n***	**%**	***n***	**%**	**Test**	***P***
Age (M ± SD)^*^	32.47 ± 9.04		31.75 ± 9.29		31.90 ± 9.44		32.48 ± 9.88		32.34 ± 9.92		32.20 ± 9.68		ANOVA/ Bonferroni	<0.01
Sex													Chi-square	<0.01
Male	19,870	92.0%	25,933	91.5%	31,790	91.7%	40,920	91.7%	44,939	91.0%	128,916	91.1%		
Female	1,735	8.0%	2,411	8.5%	2,887	8.3%	3724	8.3%	4,469	9.0%	12,664	8.9%		
Ethnic													Chi-square	<0.01
Han people	12,125	59.2%	15,884	58.9%	18,625	57.9%	23,035	56.9%	25,761	59.4%	73,578	57.5%		
Minority	8,371	40.8%	11,074	41.1%	13,534	42.1%	17,476	43.1%	17,602	40.6%	54,294	42.5%		
Marriage													Chi-square	<0.01
Single	10,152	47.0%	13,979	49.3%	17,589	50.7%	21,967	49.2%	25,754	52.1%	70,734	50.0%		
Married	10,167	47.1%	12,870	45.4%	15,396	44.4%	20,682	46.3%	21,359	43.2%	64,395	45.5%		
Widowed	92	0.4%	135	0.5%	116	0.3%	167	0.4%	194	0.4%	552	0.4%		
Divorced	1,176	5.4%	1,360	4.8%	1,576	4.5%	1,828	4.1%	2,101	4.3%	5,881	4.2%		
Education													Chi-square	<0.01
Primary school or lower	10,480	48.6%	13,203	46.6%	16,104	46.4%	21,249	47.6%	22,384	45.3%	66,301	46.8%		
Middle school	11,011	51.0%	15,027	53.0%	18,427	53.1%	23,121	51.8%	26,693	54.0%	74,457	52.6%		
University or higher	92	0.4%	111	0.4%	141	0.4%	272	0.6%	328	0.7%	791	0.6%		
Occupation													Chi-square	<0.01
Peasant	13,015	60.3%	17,248	60.9%	21,106	60.9%	26,415	59.2%	26,427	53.5%	82,747	58.5%		
Unemployed	5,258	24.4%	6,216	21.9%	6,741	19.4%	8,812	19.7%	11,510	23.3%	30,140	21.3%		
Employed, serving in the army, and others^**^	3,314	15.4%	4,880	17.2%	6,830	19.7%	9,417	21.1%	11,471	23.2%	28,675	20.3%		

* M, mean; SD, standard deviation;

** *“Others”: including retired, students, and other occupations*.

The proportion of female drug users was higher in 2015 than in any other year (*P* < 0.01), and no significant difference was found across 2011 and 2014. The proportion of identified drug users with university or higher education level increased steadily over the five studied years. There was a significant increase in the proportion of identified drug users who were employed from 2011 to 2015. Finally, the proportion of single drug users was significantly higher in 2013 and 2015 than in the other 3 years.

### Estimating prevalence of illicit drug use

Table 3 presents the estimated population sizes of any illicit drug users, heroin users, and MA users. The estimated number of any illicit drug users increased significantly from 270,388 in 2011 to a peak of 321,771 in 2014. While the estimated number of heroin users decreased from 226,435 in 2011 to 196,085 in 2015, the estimated number of MA users increased dramatically from 67,608 in 2011 to 165,179 in 2015.

**Table 3:** Prevalence rates of illicit drug use in population aged 15–64 in Yunnan (2011–15).

	**Estimated No**.			**Proportion**		**Population aged 15–64**	**Prevalence rate among 15-64 year-olds**		
	**Any drug users**	**Heroin users**	**MA users**	**Heroin users (%)**	**MA users (%)**	**(10 thousand)**	**Any drug users**	**Heroin users**	**MA users**
2011	270,388	226,435	67,608	83.74	25.00	3,356	0.81% (0.76–0.86%)	0.67% (0.63–0.73%)	0.20% (0.17–0.24%)
2012	248,979	205,912	74,007	82.70	29.72	3,370	0.74% (0.70–0.78%)	0.61% (0.57–0.65%)	0.22% (0.20–0.24%)
2013	246,196	191,572	83,339	77.81	33.85	3,373	0.73% (0.70–0.76%)	0.57% (0.54–0.60%)	0.25% (0.23–0.26%)
2014	321,771	217,463	152,919	67.58	47.52	3,427	0.94% (0.90–0.98%)	0.63% (0.60–0.67%)	0.45% (0.42–0.48%)
2015	306,958	196,085	165,179	63.88	53.81	3,449	0.89% (0.85–0.93%)	0.57% (0.53–0.61%)	0.48% (0.46–0.50%)

Estimated prevalence rates of any illicit drug use in the 15 to 64-year-old population ranged between 0.73 and 0.94%. The rate increased significantly in 2014, indicated by the corresponding 95% CIs that do not overlap with each other, and it remained at an elevated level in 2015. The estimated prevalence rate of heroin use decreased statistically significantly from 0.67% in 2011 to 0.57% in 2015. By contrast, the prevalence rates of MA use demonstrated an upward trend. In the first 3 years, the prevalence remained at lower levels, ranging between 0.20 and 0.25% with no statistically significant difference. However, it increased dramatically in the last two studied years, reaching 0.48% in 2015 (see Table 4 for more details).

**Table 4:** Completeness of identification and identified/hidden ratio of drug users in Yunnan (2011–15).

	**Completeness of identification of users**			**Identified/hidden ratio**		
	**Any drug**	**Heroin**	**MA**	**Any drug**	**Heroin**	**MA**
2011	8.0% (7.5%, 8.5%)	7.6% (7.0%, 8.2%)	6.6% (5.5%, 7.6%)	1:11.5 (10.7, 12.4)	1:12.2 (11.2, 13.3)	1:14.2 (12.0, 17.0)
2012	11.4% (10.9%, 12.0%)	10.0% (9.3%, 10.6%)	11.2% (10.2%, 12.3%)	1:7.8 (7.3, 8.3)	1:9.0 (8.4, 9.7)	1:7.9 (7.0, 8.8)
2013	14.1% (13.5%, 14.7%)	12.2% (11.5%, 12.9%)	14.2% (13.2%, 15.1%)	1:6.1 (5.8, 6.4)	1:7.2 (6.8, 7.7)	1:6.1 (5.6, 6.6)
2014	13.9% (13.3%, 14.4%)	13.2% (12.5%, 13.9%)	10.7% (10.0%, 11.4%)	1:6.2 (5.9, 6.5)	1:6.6 (6.2, 7.0)	1:8.3 (7.8, 9.0)
2015	16.1% (15.4%, 16.8%)	14.2% (13.3%, 15.2%)	13.5% (12.8%, 14.1%)	1:5.2 (5.0, 5.5)	1:6.0 (5.6, 6.5)	1:6.4 (6.1, 6.8)

### Completeness of identification of drug users and identified/hidden ratios

Completeness of identification is defined as the proportion of identified illicit drug users in the estimated population of drug users. Relatedly, the identified/hidden ratio is the ratio of the number of identified drug users to the number of hidden drug users, itself calculated by deducting the number of registered users from the total estimate). Both the rates and ratios indicate the extent and effectiveness of monitoring and capturing illicit drug users through the surveillance system.

As shown in Table 4, the completeness of the register for any illicit drug users increased from 8% in 2011 to 16% in 2015. The identified/hidden ratio changed from 1:11.5 in 2011 to 1:5.2 in 2015, meaning that for each registered drug user, there were 11.5 hidden users in 2011 and only 5.2 in 2015. The ratios for both heroin users and MA users showed a similar changing trend to that of any illicit drug users, namely, the proportions of hidden drug users decreased from 2011 to 2015.

## Discussion

This is the first estimation on the prevalence of illicit drug use in Yunnan, China using register data of illicit drug users from the police system. The prevalence rates of illicit drug use in Yunnan ranged between 0.73 and 0.94% in 2011–15. This was higher than in Le Shan City, Sichuan Province, China in 2004–07 (0.30–0.38%) (20), but close to the rates of 0.90–1.2% in some heavy drug-use regions in China in the 1990s (21, 22). The prevalence rate of illicit drug use is lower in Yunnan than in many other countries. For example, in the U.S., the National Survey on Drug Use and Health reported that 15.3% of the population has used illicit drugs in the past year (23). In 2007, 13% of Australians aged 14 years and above had used an illicit drug at least once in the previous 12 months (24). The global prevalence rates of illicit drug use remained stable at 5.2% from 2011–15 (10).

There are several potential explanations for Yunnan's illicit drug use being less prevalent than the global average. First, illicit drugs or categories of illicit drugs vary between countries and even vary between years in the same country. In the U.S., for example, there were 10 categories of illicit drugs in 2014 (marijuana, cocaine (including crack), heroin, hallucinogens, inhalants, MA, and, where misused, prescription pain relievers, tranquilizers, stimulants, and sedatives) but only seven categories in 2015 (with marijuana, cocaine (including crack), and heroin removed from the list) (25). Second, while marijuana is the most commonly used drug in many countries, it is rarely used in Yunnan. According to the World Drug Report 2017, the global prevalence rate of marijuana use in 2015 was 3.8%, while the rate in North America reached 12.4% (10). Nine percent of Australians aged 14 years or older used cannabis in the previous year in 2007 (24). By contrast, only 217 marijuana users were identified in Yunnan from 2011 to 2015, accounting for 0.15% of all identified users. In Yunnan, the most commonly used drug is heroin.

It should be noted that while the global prevalence rate remained stable from 2011 to 2015 (10, 26), illicit drug use in Yunnan increased from 0.81% in 2011 to 0.94% in 2014, and remained elevated in 2015. The increase in illicit drug use in Yunnan has mainly been caused by the emergence and spread of MA, which is relatively new in Yunnan, having only been seized for the first time in 1991 and then not again until 1996 (27). Many local users were unaware that MA is addictive or harmful due to the absence of or only mild physical withdrawal symptoms (28).

MA use in Yunnan was found to have substantially grown in recent years, with the number of identified MA users increasing by 398.5% from 2011 (4,459 users) to 2015 (22,229), and the estimated prevalence rate rising from 0.20 to 0.48% over this five-year period. This trend is also found in many other parts of the world. According to the World Drug Report 2017, the use of amphetamines, particularly MA, is perceived to be increasing in many sub-regions, including North America, Oceania, and most parts of Asia (10). In the U.S., the annual prevalence of MA use among the general population aged 15–64 remained stable between 0.5 and 0.6% from 2010 to 2013 (29), before increasing to 0.8% in 2015 (10).

Yunnan's geographic location could be a key contributor to increased MA use in the province. Since it is adjacent to Southeast Asia and lies in the Mekong region, MA is very easy to obtain. Southeast Asia experienced an MA epidemic from around 1997 and peaking in 2000–01, and after that MA trafficking and use was still increasing in parts of the Mekong region (30). With no effective treatments currently available for MA use disorders, MA could become the most widely used illicit drug in Yunnan, resulting in higher illicit drug use prevalence overall and substantial drug-related harms in the near future.

Heroin is currently the most commonly used illicit drug in Yunnan, with a higher prevalence rate than in other areas. From 2011 to 2015, heroin users accounted for more than half of all illicit drug users in both the identified and estimated population. In Yunnan's 15 to 64-year-old population, the estimated annual prevalence rates of heroin use ranged between 0.67% (2011) and 0.57% (2013, 2015), much higher than the global average of 0.37% in 2014 and 2015 (10, 26). In Europe, the average prevalence of high-risk opioid use among 15 to 64-year-olds was estimated at 0.4% in 2014 (31). Annual rates of heroin use are even lower in the U.S., recorded at 0.2% among individuals aged 12 or older, in 2011 and 0.3% in 2012–15 (25). The high prevalence of heroin use in Yunnan may have severe consequences, since opioids, including heroin, have been the most harmful illicit drug in the world, responsible for a great proportion of premature deaths among drug users (10). Globally, opioid use disorders have the heaviest disease burden of any drug use disorder (26).

We did find a recent drop in the prevalence rate of heroin use in Yunnan, which decreased from 0.67% in 2011 to 0.57% in 2015. This decline is consistent with the falling number of MMT clients in Yunnan in recent years (see Table A1). The reduced prevalence of heroin use may be attributed to the integrated measures, including MMT services, provided by the government (9). The increase in the identified/hidden ratio of users, from 1:11.5 in 2011 to 1:5.2 in 2015, also reflects a stricter control by the police and heavier punishments of illicit drug use.

However, the number of MMT clients under treatment in Yunnan was less than 10% of the province's estimated heroin users in each year of the study. Unmet treatment needs are common among substance users. In the U.S. in 2016, among people aged 12 or older who needed substance use treatment, only 10.6% (2.2 million/21.0 million) had been treated in the last year (32). For comparison, a study in Italy found that 50% of people with comorbid severe mental illness and substance use disorders received treatment for drug use (33). Possible barriers to treatment utilization may include the social stigma attached to drug users in China, the inconvenience of receiving the MMT service, and for the cost to users of the daily MMT dosage (34, 35).

The CRM model estimates population size based on details of the identified sample and several assumptions. Its first assumption, termed the closed population assumption, is that there is no emigration from and immigration into the target population during the study period. Yunnan is a large province, with a population (as of 2015) of 47 million^1^ and an area of 394,100 square kilometers^4^, so the immigration or emigration of drug users could be relatively rare. In addition, the sampling period of 1 year or longer is common in estimating population size with the CRM (14, 36–38). Within such a period, there are relatively few new drug users (including new immigrants) and drug users who die or abandon drugs (emigration), so there is likely little impact on the estimate.

The second assumption, termed the independence assumption, is that a drug user being identified has no effect on the probability of them being identified again. In this research, it is difficult to determine whether and how the assumption is violated. On the one hand, drug users who have been identified may modify their behaviors to avoid being identified again, thus leading to fewer users being identified and a smaller f_2_. In Equation (1), a smaller f_2_ would lead to a smaller N_all_, namely, underestimation of the target population. On the other hand, the police may monitor identified drug users more closely, thus increasing the probability of re-identification and, in turn, leading to overestimation of the population size.

The third, and most important, assumption in the CRM estimation is correct matching. In our research, each capture or identification was recorded with the drug user's unique identification number in the system, so matching was straightforward and easy.

There was an “Anti-Drug Battle in One-hundred Cities” in China from October 2014 to March 2015, during which the police were required to find as many drug users as possible. Consequently, more drug users than usual were captured and registered in late 2014 to early 2015, which may have caused overestimation the drug user population size.

Comorbidity is common in drug users, and in recent years, the prevalence of psychiatric disorders associated with substance use has become a matter of great concern (2). According to Hunt et al. (39), compared with non-users, illicit drug user are five times more likely to have bipolar disorder. A national survey conducted in 2008 in Italy found that among people with comorbid severe mental illness and substance use disorders, 26 and 21% suffered from 12 month dependence on alcohol and on any other substance, respectively (33). One limitation of our study is that we do not estimate the prevalence of comorbidity of substance use and mental disorders. In addition, this research focused only on illicit drug users and did not estimate the percentage of people with problem drug use or drug dependence. Such considerations are important for assessing treatment needs and operating an effective harm reduction program.

By using Zelterman's estimator, we may have underestimated the population size of drug users. There are heterogeneities of identification probability among drug users: for instance, heavier drug users or drug users with poor economic conditions may have a higher probability of identification. In most cases, the heterogeneities are unobserved, and ignoring them will lead to underestimation of the population size (15). For example, in an earlier study of illicit drug use in Bangkok in 2001, Zeltsman's estimator yielded an estimated MA user population of 33,664, compared to 34,076 using a non-parametric mixture maximum likelihood estimator. For heroin users, the discrepancy in population size between the Zelterman's estimator (12,796) and a non-parametric mixture maximum likelihood estimator (17,267) was even greater (15).

However, a nonparametric maximum likelihood estimator can sometimes produce obviously spurious results, which should be considered with great caution, whereas Zelterman's estimator provides a robust estimate (40). Overall, Zelterman's model could be the more suitable, though it may have underestimated the population size of drug users in Yunnan in our study.

To respond to the ever-increasing number of drug users in Yunnan, a more effective preventive strategy is urgently needed. This should include a public education campaign to stop MA use before it starts; improvements to MMT, such as providing take-home MMT and allocating more funding for MMT services; as well as persistence of the drug supply reduction strategy.

## Data accessibility

The datasets generated and/or analyzed during the current study are available from the first author (email: 515477781@qq.com).

## Ethics statement

The whole study was approved by the Institutional Review Board of Yunnan Institute for Drug Abuse. Data were extracted from the drug user register database, deliberately excluding personal information (name and ID). This research is a secondary data analysis, and no informed consent was required.

## Author contributions

WH designed the study. PW and XL supervised the study. JS analyzed the data. GZ and HJ wrote and revised the manuscript.

### Conflict of interest statement

The authors declare that the research was conducted in the absence of any commercial or financial relationships that could be construed as a potential conflict of interest.

## Footnotes

^1^*National Bureau of Statistics of the People's Republic of China*. Available online at http://data.stats.gov.cn/easyquery.htm?cn=E0103&zb=A030804&reg=530000&sj=2015

^2^*XinhuaNet, Illicit drug use and seizures in Yunnan*. Available online at: http://news.xinhuanet.com/local/2015-06/25/c_1115722958.htm

^3^*Yunnan Institute for Drug Abuse, Summary of Methadone Maintenance Treatment Implementation for 10 Years in Yunnan*. Available online at: http://www.kmyida.com.cn/show.aspx?model=content&id=489

^4^*People's Government of Yunnan Province Profile*, Available online at: http://www.yn.gov.cn/yn_yngk/index.html

## Appendix

**Table A1:** No. of MMT clinics and clients under treatment in China and Yunnan (2011–15).

	**No. of MMT clinics**		**No. of MMT clients**	
	**China**	**Yunnan**	**China**	**Yunnan**
2011	738	120	201,300	19,177
2012	756	141	208,400	20,720
2013	763	152	201,700	20,777
2014	767	166	184,000	20,079
2015	785	171	167,500	19,106

*Source: Yunnan Methadone Maintenance Treatment and Clean Needle/Syringe Exchange Program Secretariat*.
